# Editorial: Functional Eye Diseases: Visual Deficits and Rehabilitation

**DOI:** 10.3389/fnins.2022.842767

**Published:** 2022-02-10

**Authors:** Jiawei Zhou, Krista Kelly, Zhikuan Yang, Minbin Yu, Benjamin Thompson

**Affiliations:** ^1^State Key Laboratory of Ophthalmology, Optometry and Vision Science, School of Ophthalmology and Optometry, Eye Hospital, Wenzhou Medical University, Wenzhou, China; ^2^Retina Foundation of the Southwest, Dallas, TX, United States; ^3^Aier School of Ophthalmology, Central South University, Changsha, China; ^4^State Key Laboratory of Ophthalmology, Zhongshan Ophthalmic Center, Sun Yat-sen University, Guangdong Provincial Key Laboratory of Ophthalmology and Visual Science, Guangzhou, China; ^5^School of Optometry and Vision Science, University of Waterloo, Waterloo, ON, Canada; ^6^Centre for Eye and Vision Research, Shatin, Hong Kong SAR, China

**Keywords:** visual function, eye disease, visual rehabilitation, neuroscience, psychophysics

Normal vision is vulnerable to disorders, diseases, or injuries affecting any stage of the visual pathway that extends from the anterior surface of the eye to high-level regions of the cerebral cortex. Therefore, vision disorders can involve complex patterns of perceptual changes that may not be caused by pathology within the eye itself. We use the term “functional eye disease” to capture the broad range of conditions that can affect sight. The development of new methods for quantifying a range of visual functions is critical to enable the monitoring, prevention, diagnosis, and treatment of functional eye diseases. Novel techniques for harnessing neuroplasticity to improve cortical processing of visual information are also of significant interest in vision science, both as vision rehabilitation tools and as methods for exploring the neural mechanisms that govern visual perception. This Research Topic, “Functional Eye Diseases: Visual Deficits and Rehabilitation,” consisting of a collection of 19 fundamental and clinically oriented research articles, advances knowledge of functional eye disease detection, measurement, and rehabilitation.

Amblyopia is a developmental vision disorder caused by abnormal visual experience during early life. Deficits in amblyopia involve monocular and binocular vision, and affect both the spatial and temporal perceptual domains. Hu et al. demonstrated that amblyopia involves temporal processing deficits by reporting a flattened temporal window of visual processing, which is likely to be independent of spatial vision deficits. Hou and Acevedo Munares measured feature counting dichoptically and found an impaired ability to quickly redirect attention in amblyopia, an effect that was linked to interocular suppression. They also found different patterns of deficits in anisometropic and strabismic amblyopia, suggesting that different patterns of visual deficits are associated with amblyopia of different etiologies. Binocular treatments have the potential to change traditional approaches to amblyopia therapy. Liu, Chen et al. proposed a training strategy that focused on reducing signal threshold contrast in the amblyopic eye under a constant and high noise contrast in the fellow eye. This paradigm can encourage the amblyopic eye to cooperate more actively with the fellow eye, and better simulate normal binocular viewing conditions, in which the fellow eye exerts constant strong inhibition over the amblyopic eye. To study the underlying mechanism of impairment in amblyopia, Dai et al. used resting state fMRI (rs-fMRI) to examine effective connectivity in the brains of children and young adults with unilateral amblyopia. They found decreased effective connectivity between the left/right primary visual cortex and higher-order vision-related brain regions. Overall, their results suggested that amblyopia has a greater impact on cortical feedback than feedforward pathways.

Myopia is one of the leading causes of visual impairment worldwide. Wei et al. evaluated the effects of myopia on peripheral motion detection. They found that spatial frequency, speed, and quadrant of the visual field could significantly affect peripheral motion detection thresholds. Orthokeratology (Ortho-K), the use of specially designed contact lenses that temporarily reshape the cornea, has become one of the most common techniques to correct refractive error and control myopia progression. Xu, Tao et al. discovered that Ortho-K treatment increased children's blur detection sensitivity, which may have contributed to their good visual acuity. Liu, Wu et al. established the time course of subjective visual function changes by measuring orientation discrimination thresholds (ODT) during the first month of orthokeratology treatment in myopic children. They found that the variance in the ODT time course was not associated with axial length growth or age. Compared to multifocal soft contact lenses (MFCL, i.e., another optical device for myopia treatment), Jiang et al. found that axial length elongation in the Ortho-K group was significantly smaller than that in MFCL group, which might be related to relative corneal refractive power shift (RCRPS). Smaller RCRPS with a spatial distribution closer to the center of the cornea may enable more effective myopia prevention and control. Ye et al. found that short-term exposure to supra-threshold high-frequency flicker enhanced important aspects of spatial contrast sensitivity, which are negatively correlated with the degree of myopia. The mechanisms may involve reduced suppressive interactions between the magnocellular and parvocellular pathways and arrest of axial elongation by flicker. This finding could lead to new interventions for mild myopia.

Intermittent exotropia (IXT) is the most common form of exotropia in children and adolescents. Peng et al. measured fusional vergence amplitude, sensory fusion, and accommodative flexibility of adolescent IXT after successful surgery. They concluded that binocular function continued to improve postoperatively in adolescents with IXT, while no significant correlations were found between binocular functions postoperatively and ocular alignment stability. Using rs-fMRI of the brain, He et al. analyzed the amplitude of low-frequency fluctuations and functional connectivity in patients with IXT. They found that IXT patients had abnormalities in brain areas related to vision and eye movements. These results bare upon the neuropathological mechanisms of vision and ocular motor impairments in IXT patients.

Vision plays an important role in the quality of life of older adults. Since the aging population is growing, it is important to understand how age affects visual perception. Xia et al. found that the ability to discriminate stimulus orientation and motion direction is decreased in older adults. This effect could be related to age-related impairments in visual cortex function. Lin et al. showed that the association between poorer stereopsis and lower inhibitory control in older adults might be caused by central nervous system impairment that affects the processing of binocular disparity and antisaccades.

Functional eye disease is also a useful tool for investigating visual development. Macneill et al. used a new method to study the development of visuomotor responses (responses to moving targets or laser spots) in normal and visually deprived cats. As expected, visual deprivation significantly impaired spatial vision; however, visuomotor function was preserved. This indicates that the neuronal populations that support spatial vision and visuomotor function are separate. By measuring the contrast sensitivity function and spatial sweep visual evoked potentials (sVEP) with vertical and horizontal sinewave gratings, Gu et al. found that participants with astigmatism exhibited marked vertical-horizontal resolution disparities. This suggests that meridian-specific partial deprivation in early life leads to monocularly asymmetric development of spatial vision in humans.

Visual function assessment is important to diagnose, evaluate, and treat eye diseases. Therefore, the development of efficient, accurate, and convenient tests is highly desirable. Contrast sensitivity is an important indicator for assessing functional vision. Zhuang et al. found that eye tracking technology can be used to accurately quantify contrast sensitivity in adults. Future application of the technique to infants and non-verbal individuals is planned. Zheng et al. found that binocular accommodative facility and cut-off spatial frequency were sensitive to visual fatigue, which disrupted the ability of adult subjects to encode visual details. Xu, Lesmes et al. developed a multi-module active learning framework called the qVFM. They used two switching methods, the distribution sampling method (DSM) and parameter delivery method (PDM) to evaluate the performance of the qVFM method in mapping the light sensitivity visual field maps (VFMs) of simulated patients. The results showed this method can provide accurate and efficient assessments of the light sensitivity VFM for simulated patients, which can be used to characterize residual vision of simulated ophthalmic patients. In addition, the qVFM-PDM method exhibited better performance than qVFM-DSM in detecting VF loss in simulated glaucoma. However, simulated eye disease may differ from the real effects of vision loss. Macnamara et al. conducted a systematic review that synthesized and assessed various AMD simulation methods by investigating activities of daily living and evaluating clinical validation procedures and the adaptation periods for participants. They concluded that all simulations have limitations, and the nature of the study should continue to guide the choice of simulation.

Altogether, this collection of articles highlights the vision deficits associated with functional eye diseases using both basic and clinical research methodologies. It also contains methods to evaluate and treat vision loss. Adding to the plethora of current knowledge of functional eye diseases, their deficits, and treatments, these articles will pave the way for expanding vision research and developing future preventions and interventions to curb vision loss.

## Author Contributions

All authors listed have made a substantial, direct, and intellectual contribution to the work and approved it for publication.

## Funding

JZ is supported by the National Natural Science Foundation of China Grant (NSFC31970975), the Natural Science Foundation for Distinguished Young Scholars of Zhejiang Province, China (LR22H120001), and the Project of State Key Laboratory of Ophthalmology, Optometry and Vision Science, Wenzhou Medical University (No. J02-20210203). BT is supported by the Hong Kong Special Administrative Region Government and InnoHK, NSERC Discovery Grant RGPAS-477166, CIHR Grant 390283, and CFI Grant 34095.

## Conflict of Interest

The authors declare that the research was conducted in the absence of any commercial or financial relationships that could be construed as a potential conflict of interest.

## Publisher's Note

All claims expressed in this article are solely those of the authors and do not necessarily represent those of their affiliated organizations, or those of the publisher, the editors and the reviewers. Any product that may be evaluated in this article, or claim that may be made by its manufacturer, is not guaranteed or endorsed by the publisher.

